# Integral membrane protein 2A inhibits cell growth in human breast cancer via enhancing autophagy induction

**DOI:** 10.1186/s12964-019-0422-7

**Published:** 2019-08-22

**Authors:** Cefan Zhou, Ming Wang, Jing Yang, Hui Xiong, Yefu Wang, Jingfeng Tang

**Affiliations:** 1 The State Key Laboratory of Virology, College of Life Sciences, Wuhan University, Wuhan, China; 2 National “111” Center for Cellular Regulation and Molecular Pharmaceutics, Hubei University of Technology, Wuhan, China; 3 Department of Clinical Laboratory, Renmin Hospital of Wuhan University, Wuhan, China; 4 Robert H. Lurie Comprehensive Cancer Center, Department of Medicine-Division of Hematology/Oncology, Northwestern University Feinberg School of Medicine, Chicago, USA; 5 Department of Clinical Laboratory, Hospital of Southern University of Science & Technology, Shenzhen, Guangzhou, China; 6 XiLi People’s Hospital, Shenzhen, Guangzhou, China

**Keywords:** ITM2A, Autophagy, Prognosis, Breast cancer, Up-regulated Neu-associated kinase, HUNK

## Abstract

**Background:**

Breast cancer is a life-threatening disease in females and the leading cause of mortality among the female population, presenting huge challenges for prognosis and treatment. ITM2A is a member of the BRICHOS superfamily, which are thought to have a chaperone function. ITM2A has been identified to related to ovarian cancer progress recently. However, the biological role of ITM2A in breast cancer remains largely unclear.

**Methods:**

Quantitative real-time polymerase chain reaction (qRT-PCR), western blotting assay and immunohistochemistry staining were used to analyzed the expression level of ITM2A. The patient overall survival versus ITM2A expression level was evaluated by Kaplan-Meier analysis. MTT assay, EdU incorporation assay and colony formation assay were used to evaluated the role of ITM2A on breast cancer cell proliferation. Autophagy was explored through autophagic flux detection using a confocal microscope and autophagic vacuoles investigation under a transmission electron microscopy (TEM). In vitro kinase assay was used to investigated the phosphorylation modification of ITM2A by HUNK.

**Results:**

Our data showed that the expression of integral membrane protein 2A (ITM2A) was significantly down-regulated in human breast cancer tissues and cell lines. Kaplan-Meier analysis indicated that patients presenting with reduced ITM2A expression exhibited poor overall survival, and expression significantly correlated with age, progesterone receptor status, TNM classification and tumor stage. ITM2A overexpression significantly inhibited the proliferation of breast cancer cells. By studying several autophagic markers and events in human breast cancer SKBR-3 cells, we further demonstrated that ITM2A is a novel positive regulator of autophagy through an mTOR-dependent manner. Moreover, we found that ITM2A was phosphorylated at T35 by HUNK, a serine/threonine kinase significantly correlated with human breast cancer overall survival and HER2-induced mammary tumorigenesis.

**Conclusion:**

Our study provided evidence that ITM2A functions as a novel prognostic marker and represents a potential therapeutic target.

**Electronic supplementary material:**

The online version of this article (10.1186/s12964-019-0422-7) contains supplementary material, which is available to authorized users.

## Background

Breast cancer is the most prevalent malignancy worldwide and the second leading cause of cancer death for women. In 2018 alone, the estimated number of newly diagnosed cases and deaths from breast cancer in women in the United States were 266,120 and 40,920, respectively [1]. It is a heterogeneous disease with differences in clinical, molecular and biological features and thus creates a challenge for prognosis and treatment. Despite dramatic advances in the breast cancer research setting and the diagnosis and treatment of breast cancer, the prognosis and survival for most patients, particularly those with metastases, is dramatically low [2]. With the standardization of systemic chemotherapy as the gold-standard approach for most cancer types, substantial acquired drug resistance to therapy frequently crops up and represents a major problem [3]. Important progress has been made for human epidermal growth factor receptor 2 (HER2)-positive breast cancer, which accounts for 20% of all breast cancer patients [4]. Patients with HER2-positive cancers who received trastuzumab and pertuzumab concurrently with chemotherapy followed by trastuzumab after surgery significantly improved overall survival in metastatic and adjuvant settings [5]. However, recurrence and disease progression rates remain dramatically high. Therefore, the identification of diagnostic markers and potential cellular and molecular mechanisms underlying breast cancer metastasis, as well as the development of new therapeutic strategies for improving patient survival and overall quality of life, are urgently needed [6].

Macroautophagy (hereafter referred to as autophagy) is an evolutionarily conserved stress-responsive process that degrades and recycles superfluous or potentially dangerous cytosolic entities (e.g., damaged mitochondria, invading pathogens). Autophagy is characterized by the formation of a double-membrane structure called the phagophore that recruits autophagy-related proteins and fuses with the lysosomes [7]. AMP-activated protein kinase (AMPK), a key energy sensor that regulates cellular metabolism to maintain energy homeostasis, and the mammalian target of rapamycin (mTOR), a central cell-growth regulator that integrates growth factor and nutrient signals, are the two central modulators of autophagy regulation [8]. Prevention of Ser 757 phosphorylation of ULK1 by mTOR results in ULK1 dissociation from both the AMPK and mTOR complex and then phosphorylation of ATG13 and RB1-inducible coiled-coil (1RB1CC1/FIP200), thus triggering autophagy under nutrient sufficiency [8, 9]. Inactivation of mTORC1 by amino acid starvation can activate autophagy-specific (ATG14-containing) type III phosphatidylinositol (PtdIns) 3 kinase (PIK3C3/VPS34) and induce autophagy both in vitro and in vivo [10]. Autophagy occurs frequently during tumorigenesis and cancer chemotherapy. Increasing evidence has suggested that enhancers of autophagy might prevent cancer development in premalignant lesions [11, 12].

ITM2A is a 263-amino acid type II transmembrane protein and belongs to a family of integral membrane proteins, including ITM2B and ITM2C, all of which are part of the BRICHOS superfamily. A 100-amino acid BRICHOS domain is found in the BRICHOS family proteins, which are thought to have a chaperone function [13]. There are four defined regions in the ITM2A protein, which are the hydrophobic, linker, extracellular BRICHOS and the intracellular C-terminal domains [13]. ITM2A has been demonstrated as a transcriptional target of PKA-CREB, GATA3 and PAX3 in diverse systems [14–16]. By using cDNA library subtraction, ITM2A was initially identified as a candidate marker for chondro-osteogenic differentiation [17]. ITM2A has also been reported to function in autoimmune disease and myogenic differentiation [18, 19]. Recently, the role of ITM2A in epithelial ovarian cancer has been identified [20]. However, the biological role of ITM2A in breast cancer remains largely unclear.

In the present study, we extend and highlight the role of ITM2A in breast cancer via regulation of autophagy. We found that breast cancer patients with low ITM2A expression exhibit poor overall survival and that overexpression of ITM2A significantly inhibits breast cancer cell proliferation. We also linked the protein phosphorylation modification of ITM2A by HUNK, a serine/threonine kinase significantly connected with human breast cancer overall survival and HER2-induced mammary tumorigenesis. We also showed that ITM2A is involved in autophagy in an mTOR-dependent manner.

## Methods

### Cell lines, reagents, and antibodies

Human breast cancer cell lines MDA-MB-468, BT474, SKBR-3 and normal breast epithelial cell line Hs578Bs were purchased from the Cell Center of Institute of Biochemistry and Cell Biology, Chinese Academy of Sciences (Shanghai, China). Human breast cancer cell lines MCF-7, MDA-MB-231 and human embryonic kidney cell line HEK293T were kept in our laboratory. EBSS (Hyclone) starvation was carried out to switch the culture medium from the complete medium to EBSS medium. Commercially available antibodies and dilutions used are as follows: anti-ITM2A (Proteintech, cat: 18306–1-AP, 1:1000 dilution), anti-GAPDH (Proteintech, cat: 60004–1-Ig, 1:1000 dilution), anti-LC3 (Cell Signaling Technology, cat: 12741, 1:1000 dilution), anti-P62/SQSTM1 (Boster, cat: BM4385, 1:1000 dilution), anti-Rabbit Flag (EMD Millipore, cat: PM020A, 1:1000 dilution), anti-pAMPK-T172 (Cell Signaling Technology, cat: 2535, 1:1000 dilution), anti-AMPK (Boster, cat: A30453, 1:500 dilution), anti-p4EBP1-T37/46 (Cell Signaling Technology, cat: 2855, 1:1000 dilution), anti-4EBP1 (Proteintech, cat: 60246–1-Ig, 1:1000 dilution). anti-phospho-MBP (EMD Millipore, cat: 05–429, 1:1000 dilution), anti-MBP (Proteintech, cat: 10458–1-AP, 1:1000 dilution), anti-HUNK (Invitrogen, cat: PA5–28765, 1:1000 dilution).

### Plasmids construction, cell culture and transfection

DNA fragments encoding ITM2A amplified by PCR was gift from professor Jiahuai Han (Xiamen University). The PCR products of ITM2A and HUNK were both cloned into pCMV-3 × Flag. ITM2A was also cloned into pGEX-4 T1 plasmid. The nucleotide sequences of all constructs were confirmed by DNA sequencing. ptfLC3 (Mammalian expression of rat LC3 fused to mRFP and GFP) was a gift from professor Tamotsu Yoshimori (Addgene plasmid # 21074). Human normal pancreatic duct epithelial cells HPD E6-C7, human breast cancer cell lines MCF-7, MDA-MB-231, MDA-MB-468, BT474, SKBR-3 and normal breast epithelial cell line Hs578Bst, as well as HEK293T were all cultured in Dulbecco’s modified Eagle’s medium (DMEM) (Gibco, USA). Culture mediums were supplemented with 10% fetal bovine serum (Gibco, USA), 100 U/ml penicillin G and 100 μg/ml streptomycin at 37 °C in a humidified incubator containing 5% CO2. siRNA to HUNK was 5′-AUAGAGAAUUUGCUACUAGAU-3′. siRNA to ITM2A was 5′-ACUCAUGCAUUUACUCUAUUG-3′ Lipofectamine 2000 Transfection Reagent (Invitrogen, USA) was used to transfect the SKBR-3 and MDA-MB-231 cell lines with the expression vector of ITM2A or HUNK according to the manufacturer’s protocols.

### The bioinformatics analysis of gene expression in breast cancer patients

The cancer genome atlas (TCGA) analysis of gene expression in breast cancer patients was previously described [6]. Fold-change analysis was performed on the two categories of samples (Normal and Tumor), followed by an unpaired t-test (unequal variance) that was performed to obtain significant gene entities. Gene microarray datasets GSE 86374 and GSE 10916 were downloaded from the Gene Expression Omnibus database (https://www.ncbi.nlm.nih.gov/geo/). Bc-GenExMiner v4.2 (http://bcgenex.centregauducheau.fr/BC-GEM/GEM-Accueil.php?js=1) were used to performed the meta-analysis for breast cancer overall survival between the five breast cancer subtypes.

### RNA extraction and quantitative real-time polymerase chain reaction (qRT-PCR)

RNA extraction and quantitative real-time polymerase chain reaction (qRT-PCR) were previously described [6]. The mRNA expression level for each sample was normalized to the expression of GAPDH using the 2 -ΔΔct method [21]. The following primer sequences were used for qRT-PCR: ITM2A, (forward) 5′- CGCGGCAAGACGTGGAG − 3′ and (reverse) 5′- CCACTCGCCAGTTTGCCA -3′; GAPDH, (forward) 5′- AGCCACATCGCTCAGACAC − 3′ and (reverse)5′- GCCCAATACGACCAAATCC − 3′;

### Immunoprecipitation

SKBR-3 and HEK293T cells were lysed with RIPA lysis buffer (50 mM Tris-HCl pH 7.4, 150 mM NaCl, 1% Triton X-100, 10 mM NaF, 1 mM EDTA and proteinase inhibitor cocktail (Roche Applied Sciences) and halt phosphatase inhibitor cocktail (Thermofisher Scientific)) for 30 min on ice after washing twice with phosphate-buffered saline (PBS). The lysates were pretreated with protein A/G beads (Santa Cruz Biotechnology) and then centrifuged at 12,000×g for 10 min at 4 °C. The supernatants were incubated with ITM2A or HUNK antibodies overnight at 4 °C and subsequently incubated with secondary antibodies for 2 h. After that, the lysates were incubated with protein A/G agarose beads (Santa Cruz Biotechnology) for 2 h at 4 °C. The beads were washed twice with PBS buffer, twice with RIPA lysis buffer and twice with TBST buffer to fully eluting the impurity. Protein concentration was measured by Biorad Protein Assay, and the immunoprecipitate were subjected to western blot.

### Western blotting

Western blotting were previously described [6]. Briefly, cell lysates were boiled in 2 × SDS loading buffer for 10 min at 98 °C and then loaded on SDS-PAGE gels. Gels were then blotted on 0.45 μm polyvinylidene fluoride membrane (PVDF) (Millipore). The signals of the protein in the PVDF membranes were detected using supersignal west pico plus (Invitrogen, cat: 34580) after incubated with primary antibodies and secondary antibodies.

### MTT assay

MTT assay was performed as described previously [22]. Briefly, human breast cancer SKBR-3 and MDA-MB-231 cells (1 × 10^3^ cells/well) were seeded into 96 - well plates, the cells were then stained at the indicated time points with 100 μl sterile MTT dye (0.5 mg/ml, Sigma, USA) for 4 h at 37 °C after ITM2A transfected and later 48 h culture. After adding 150 μ l DMSO (Sigma), the number of viable cells was assessed by measurement of the absorbance at 450 nm by a microplate reader. All experiments were performed in triplicates.

### Colony formation assay

Human breast cancer SKBR-3 and MDA-MB-231 cells transfected with ITM2A were seeded into 12 - well plate and incubated with complete medium at 37 °C for 2–3 weeks. Then, the cells were fixed with 4% paraformaldehyde and stained with 2% crystal violet. The images were obtained using an inverted microscope.

### 5-Ethynyl-20-deoxyuridine (EdU) incorporation assay

The BeyoClick™ EdU Cell Proliferation Kit with Alexa Fluor 555 was purchased from Beyotime (Shanghai, China). SKBR-3 and MDA-MB-231 cells transfected with ITM2A were plated in 24 wells and cultured for 48 h, and then fixed using 4% paraformaldehyde and stained with DAPI after incubation with 50 mM EdU solution for 2 h. The EdU labeled cells were photographed under an Olympus FSX100 microscope (Olympus, Tokyo, Japan).

### Electron microscopy assay

SKBR-3 cells were washed in PBS twice and fixed in 2.5% glutaraldehyde at 4 °C overnight, and then the cells were sent to Servicebio (Wuhan) for electron microscopy assay services.

### Confocal microscopy

SKBR-3 cells transfected with the appropriate plasmids were grown on glass chambers at 60% density and incubated for 42 h. Cells were then fixed with 4% paraformaldehyde in PBS for 10 min. After cells were permeabilized with 0.5% Triton X-100 in PBS for 15 min, cells were blocked with 10% goat serum and then incubated with a 1:100/1:200 dilution of corresponding primary antibodies overnight at 4 °C and a 1:100 dilution of fluorescence-labeled secondary antibodies diluted in immunostaining buffer for 2 h. DAPI was used for nuclei staining. Cells were examined with a confocal laser-scanning microscopy (Leica SP8, Wetzlar, Germany) using a 63× oil immersion objective.

### Immunohistochemistry

Immunohistochemistry was performed as described previously [23]. Briefly, the antibody against ITM2A (Proteintech) was tested on sections from a human tissue array that contain formalin-fixed paraffin-embedded human breast cancer tissues (*n* = 50) and para-carcinoma tissues (*n* = 36) (Servicebio, cat: IWLT-N-86B62 BC-1602). To quantify the status of ITM2A protein expression in those groups, the intensity of the ITM2A immunoreaction was scored as follows: 0, none; 1, weak; 2, moderate; and 3, intense.

### In vitro kinase assay

Flag-HUNK expressed in 293 T cells in the nutrient rich or EBSS starvation conditions were immunoprecipitated using anti-Flag antibody. The immunoprecipitates were then washed with RIPA buffer twice, followed by washing with kinase assay buffer (25 mM HEPES, pH 7.5, 10 mM MgCl2, 50 mM NaCl, 1 mM DTT, 0.5 mg/ml BSA, 250 μM Na3VO4, 50 mM NaF, 1 mM EDTA, 0.2 mM AMP). And then the immunoprecipitated HUNK beads were incubated with purified recombinant GST-ITM2A WT, T35A mutant or MBP protein in kinase buffer containing nonradioactive 0.2 mM ATP at 30 °C for 30 min. Reactions were stopped by boiling the mixtures in SDS sample buffer, followed by SDS-PAGE and analyzed by western blotting.

### Statistical analysis

Receiver operating characteristic (ROC) curve analysis was used to evaluate the prognosis significantly of ITM2A. The area under the curve (AUC) was computed via numerical integration of the ROC curves. The Mann-Whitney test and the two-tailed *p*-value were used to compare the difference between groups. All experiments were performed independently at least three times. All statistical analysis was performed using GraphPad Prism 6.0 software (GraphPad, La Jolla, CA, USA). All data were presented as mean ± SD (standard deviation). *p* values < 0.05 were statistically significant.

## Results

### ITM2A is down-regulated in human breast cancer

**Fig. 1:**
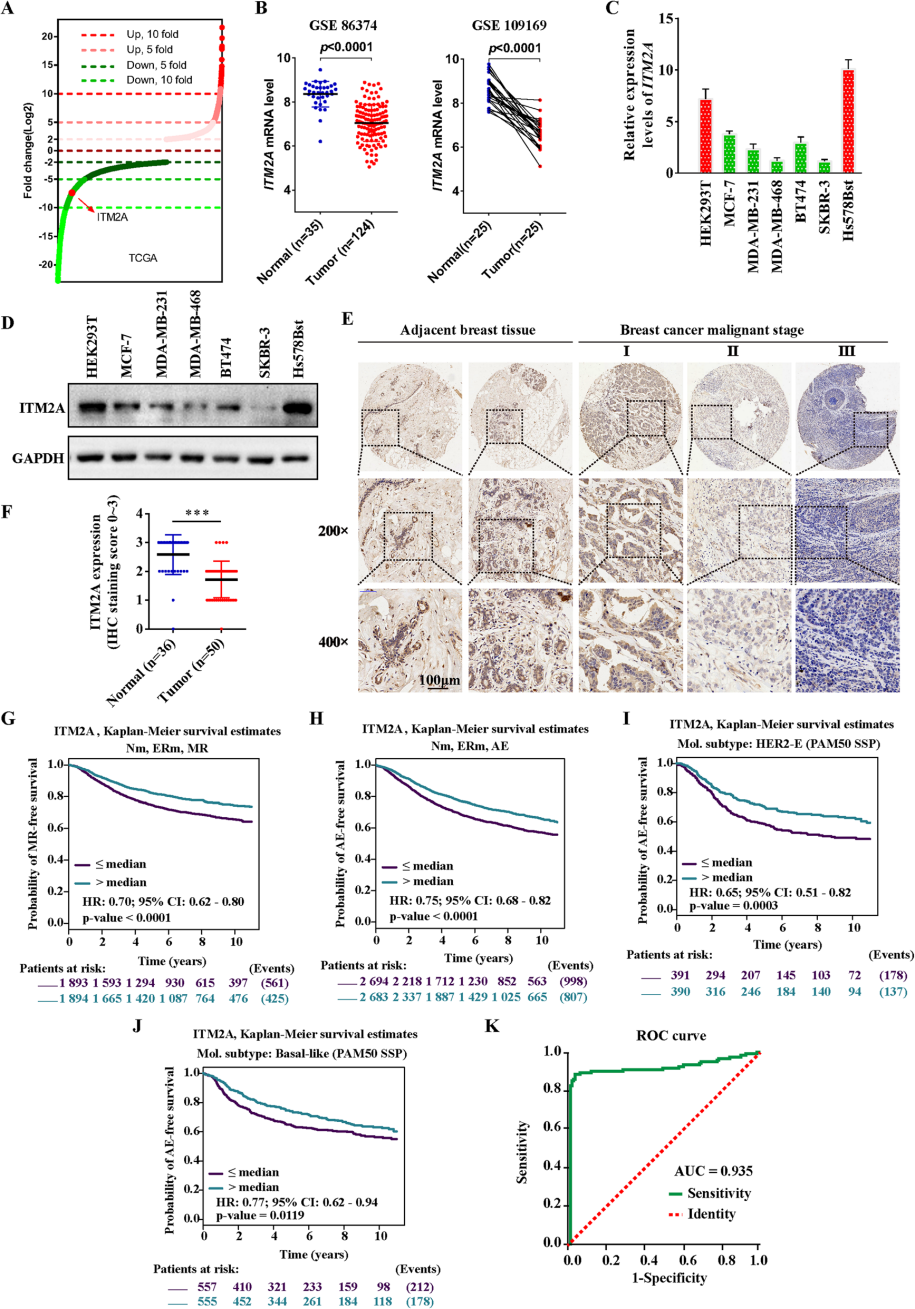
ITM2A was down-regulated and low ITM2A showed poor clinical outcomes in human breast cancer.(**a**) Distribution of fold changes illustrated in gene expression profiles for the breast cancer tissues obtained from the TCGA database. The log2 values were calculated for each sample by normalizing to read counts alone (log2 Fold Change). Gene expression analysis was performed using GeneSpringGX software. (**b**) The mRNA level of ITM2A in nonpaired (left, GSE86374) and paired (right, GSE109169) breast cancer tissues from the GEO database. (**c** and **d**) The mRNA and protein levels of ITM2A in the human breast cancer cell lines MCF-7, MDA-MB-231, MDA-MB-468, BT474, and SKBR-3 and the normal breast epithelial cell line Hs578Bst, as well as the human embryonic kidney cell line HEK293T. (**e**) Representative immunohistochemical staining images of ITM2A expression in human breast cancer tissues (I, II and III) and para-carcinoma tissues. (**f**) The immunohistochemical staining intensity of ITM2A expression in breast cancer tissues and para-carcinoma tissues. The intensity of the ITM2A immunoreaction was scored as 0, none; 1, weak; 2, moderate; and 3, intense. (**g**-**j**) Kaplan-Meier survival curves for the association between ITM2A expression and the probability of overall survival of human breast cancer patients. “Patients at risk” refers to patients who are at risk of the event occurrence, such as death or metastatic relapse. (**k**) The ROC curve of ITM2A expression in human breast cancer patients. Analysis of the ITM2A expression levels resulted in an area under the curve (AUC) value of 0.935. Data are presented as the mean ± SD. Two-tailed Student’s t-test was used. *P <  0.05; **P <  0.01; ***P <  0.001

Previously, using a meta-analysis of publicly available mRNA expression data, we identified single-gene prognostic biomarkers in breast cancer [6]. Through genome-wide expression analysis, we found that the mRNA level of ITM2A was 7.40-fold down-regulated in breast cancer tissues (*n* = 1101) compared with that in normal tissues (*n* = 139) from the TCGA database of breast cancer (Fig. 1a). Similar expression patterns of ITM2A were found in the gene microarray datasets GSE 86374 and GSE 10916 (Fig. 1b). We next verified the expression of ITM2A in a panel of human breast cancer cell lines, including MCF-7, MDA-MB-231, MDA-MB-468, BT474 and SKBR-3, and the normal breast epithelial cell line Hs578Bst, as well as the human embryonic kidney cell line HEK293T. The results showed a lowered expression of ITM2A in human breast cancer cell lines (Fig. 1c). To examine whether the observed decrease in ITM2A mRNA expression was translationally relevant, we examined ITM2A protein expression in the cell lines mentioned above and utilized a commercial breast cancer tissue array. Western blotting analysis revealed a similar decrease in ITM2A expression in human breast cancer cell lines (Fig. 1d). Immunohistochemical (IHC) analysis also revealed that ITM2A expression levels were significantly decreased in the advanced stages of breast cancer compared to those in adjacent normal tissues (Fig. 1e and f).

### Low ITM2A expression is associated with clinicopathological features and poor prognosis in human breast cancer patients

We next investigated the relevance of the ITM2A expression level to the clinicopathological features of breast cancer patients from the TCGA database. We found that decreased expression of ITM2A significantly correlates with age, progesterone receptor (PR) status, TNM classification and tumor stage but not with estrogen receptor (ER) status and human epidermal growth factor receptor 2 (HER2) levels (Table 1). We next set out to identify the prognostic potential of ITM2A expression in breast cancer patients and to investigate whether ITM2A expression is related to breast cancer patient outcome. We carried out a series of univariate Cox proportional hazards model analyses and Kaplan-Meier analyses of the overall survival using the Bc-GenExMiner v4.2 database [24]. According to the nodal status and ER status and the clinical events of metastatic relapse (MR) and any event (AE) (first pejorative event represented by any relapse or death), a total of 18 combined populations (pools) were analyzed (Table 2). The AE (for Nm, ERm and AE: *p* <  0.0001, HR = 0.75, 95% CI = 0.68–0.82) and MR (for Nm, ERm and MR: *p* <  0.0001, HR = 0.70, 95% CI = 0.62–0.80) data indicated that low ITM2A expression is associated with poor prognosis for breast cancer. Moreover, the results indicated that decreased ITM2A expression in nodal-negative patients exhibits more significant prognostic significance than nodal-positive patients (for N-, ER+ and MR: *p* value < 0.0001, HR = 0.58, 95% CI = 0.46–0.74, NP = 1347 and N+, ER+ and MR: p value = 0.3732, HR = 0.88, 95% CI = 0.66–1.17, NP = 665). Simultaneously, the Kaplan-Meier analysis showed that lowered expression of ITM2A exhibits poor outcomes in breast cancer patients (Fig. 1g and h**;** Additional file 1: Figure S1 A-L). Additionally, we found that decreased expression levels of ITM2A in patients classified as HER2-E (HER2-enriched) and basal-like subtypes showed reduced overall survival time (Fig. 1i and j**;** Additional file 1**:** Figure S2 A-C). Furthermore, we generated receiver operating characteristic (ROC) curves and found that the ITM2A mRNA level in breast cancer tissues substantially differs from that in normal tissues, with an area under the curve (AUC) value of 0.935 (95% CI: 0.923–0.948) (Fig. 1k). Using the optimal threshold value of 0.863, the sensitivity and specificity values were 0.890 and 0.028, respectively, to identify a patient with breast cancer, indicating that ITM2A serves as an excellent breast cancer marker.

**Table 1:** Clinicopathologic characteristics of breast cancer patients with different ITM2A expression from the TCGA database

Characteristic	No. (%) of patients			Correlation	
	Total	ITM2A high	ITM2A low	Chi-square	*p* value
Age in years	1208			6.048	0.0139
≤ 60		285	362		
> 60		208	353		
ER status	1145			3.016	0.0824
Positive		382	504		
Negative		96	163		
PR status	1142			6.734	0.0095
Positive		340	427		
Negative		136	239		
HER2 level				2.726	0.2559
1+		122	160		
2+		75	134		
3+		39	57		
T classification	1205			12.67	0.0018
T1		145	164		
T2		256	443		
T3/T4		92	105		
N classification	1185			8.345	0.0394
N0		261	300		
N1		220	191		
N2		68	64		
N3		49	32		
Stage	1199			14.62	0.0022
I		92	110		
II		254	432		
III		134	141		
Other		11	25		

*ER* estrogen receptor, *PR* progesterone receptor, *HER2* receptor tyrosine-protein kinase erbB-2

**Table 2:** Target prognostic analysis for the ITM2A expression levels in 18 pools corresponding to combinations of populations (ER and Nodal status) and clinical event criteria (MR or AE)

Nodal status	Estrogen receptor status	Event status	*p*-value	HR	95% CI	No. patients	No. events
Nm	ERm	AE	< 0.0001	0.75	0.68–0.82	5377	1805
Nm	ERm	MR	< 0.0001	0.7	0.62–0.80	3787	986
N-	ERm	MR	< 0.0001	0.6	0.49–0.72	1848	446
Nm	ER-	AE	< 0.0001	0.65	0.55–0.76	1517	592
N-	ER+	MR	< 0.0001	0.58	0.46–0.74	1347	302
N-	ERm	AE	< 0.0001	0.71	0.61–0.83	2384	710
Nm	ER+	AE	< 0.0001	0.78	0.69–0.87	3813	1202
Nm	ER-	MR	< 0.0001	0.63	0.51–0.79	1058	340
N-	ER+	AE	0.0001	0.69	0.57–0.83	1727	488
Nm	ER+	MR	0.0001	0.73	0.62–0.86	2700	641
N+	ERm	AE	0.0033	0.79	0.67–0.92	1483	618
N+	ER-	AE	0.0125	0.7	0.53–0.93	444	218
N-	ER-	MR	0.0301	0.68	0.48–0.96	483	142
N-	ER-	AE	0.0431	0.75	0.57–0.99	634	217
N+	ER-	MR	0.097	0.74	0.52–1.06	310	127
N+	ER+	AE	0.0997	0.84	0.69–1.03	1030	399
N+	ERm	MR	0.1606	0.85	0.68–1.06	983	323
N+	ER+	MR	0.3732	0.88	0.66–1.17	665	195

*N (+, −, m)* nodal status (+: positive, −: negative, m: mixed); estrogen receptor status (+: positive, −: negative, m: mixed), *MR* metastatic relapse, *AE* first pejorative event represented by any relapse or death, *HR* hazard ratio (values are rounded to 2 decimal places), *95% CI* 95% confidence interval (values were rounded to 2 decimal places)

### Overexpression of ITM2A inhibits the proliferation of breast cancer cells

**Fig. 2:**
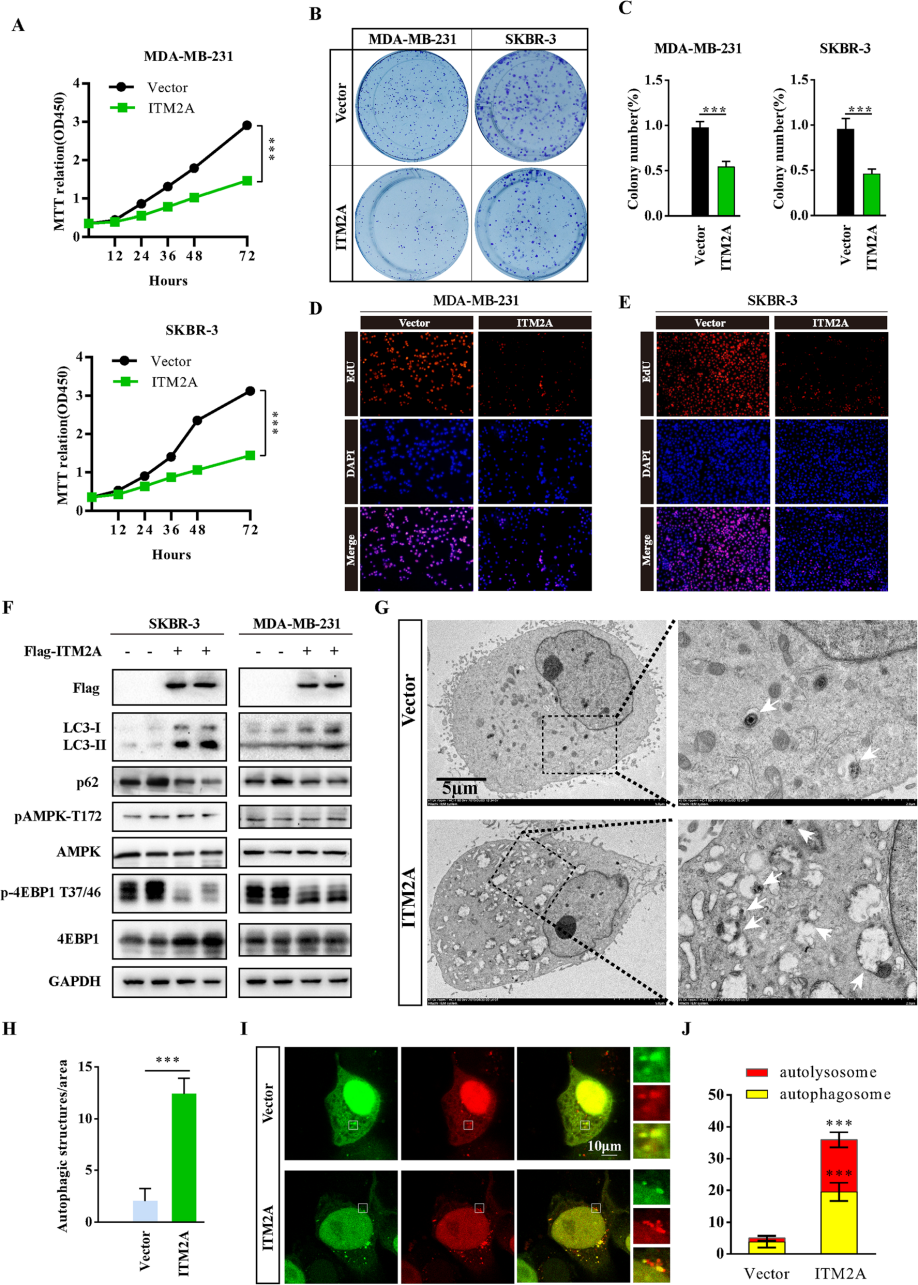
Overexpression of ITM2A inhibits breast cancer proliferation through autophagy induction.(**a**) The growth curve of MDA-MB-231 and SKBR-3 cells transfected with empty vector or ITM2A expression plasmids. (**b** and **c**) Colony formation assays were performed for MDA-MB-231 and SKBR-3 cells transfected with empty vector or ITM2A plasmids. (**d** and **e**) DNA synthesis of MDA-MB-231 and SKBR-3 cells transfected with ITM2A vectors was assessed by EdU assays. (**f**) Western blotting analysis in SKBR-3 and MDA-MB-231 cells transfected with empty vector and ITM2A expression vector using the indicated antibodies. (**g** and **h**) Representative electron microscope images of autophagosomes or autolysosomes of SKBR-3 cells transfected with empty vector and ITM2A expression vector. Both low- and high-power (zoom) images are displayed. White arrows indicate autophagic structures. The number of autophagic structures was quantified (*n* = 10). (**i** and **j**) Representative confocal images of mRFP-GFP-LC3 puncta in SKBR-3 cells transfected with the ITM2A vector. The numbers of cells showing accumulation of yellow or red puncta were quantified (*n* = 10). Data are presented as the mean ± SD. Two-tailed Student’s t-test was used. *P <  0.05; **P <  0.01; ***P <  0.001

To investigate the role of ITM2A in the proliferation of breast cancer cells, we generated the growth curve of MDA-MB-231 cells after ITM2A overexpression using 3-[4,5-dime-thylthiazol-2-yl]-2,5 diphenyl tetrazolium bromide (MTT) assays. The results showed that ITM2A overexpression significantly inhibited MDA-MB-231 cell growth, and similar results were found in SKBR-3 transfected cells (Fig. 2a). Moreover, the colony formation activity of MDA-MB-231 and SKBR-3 was inhibited after ITM2A expression (Fig. 2b and c). Furthermore, overexpression of ITM2A markedly decreased DNA synthesis in MDA-MB-231 and SKBR-3 cells by the 5-ethynyl-20-deoxyuridine (EdU) incorporation assay (Fig. 2d and e). These results indicated that ITM2A plays an essential role in breast cancer proliferation.

### ITM2A promotes autophagy flux in an mTOR-dependent manner in breast cancer cells

It was previously reported that ITM2A expression interferes with autophagic flux by interacting with vacuolar ATPase (v-ATPase) in HEK293T cells [14], however, in the context of tumor cells, whether ITM2A could be modified and the mechanisms of the regulation and modification need to be verified, especially in human breast cancer cells. To test the effect of ITM2A on autophagy flux in breast cancer cells, we detected the protein levels of LC3-II and p62 through western blotting assays, autophagic vacuoles by electron microscopy, and the autolysosome formation using the tandem reporter mRFP-GFP-LC3. The results revealed that ITM2A increased the level of LC3-II and decreased the p62 level (Fig. 2f). Moreover, we found markedly increased autophagic vacuoles in ITM2A transfected SKBR-3 cells (Fig. 2g and h). Additionally, ITM2A overexpression increased the number of red-only LC3 puncta, which represents autolysosomes (Fig. 2I and J). We also checked the effect of ITM2A knockdown on human embryonic kidney 293 T cells, which present high levels of ITM2A. The results showed that ITM2A depletion decreased the level of LC3-II and increased the level of p62 (Additional file 1: Figure S3). Taken together, these data indicated that ITM2A promotes autophagy flux in breast cancer cells.

We then asked whether ITM2A modulated upstream autophagy-regulatory signaling pathways, such as inhibition of mTOR activity or promotion of AMPK, two main signaling pathways that function in autophagy through directly phosphorylating ULK1 [8]. We found that ITM2A significantly inhibited the activity of mTOR, indicated by the reduced level of phosphorylated 4EBP1 at both threonine (T) 37/46, whereas there was no influence on the AMPK pathway (Fig. 2f). These data indicated that ITM2A promotes autophagy flux in an mTOR-dependent manner.

### ITM2A is phosphorylated at T35 and the phosphorylation status of ITM2A is required for autophagy induction

**Fig. 3:**
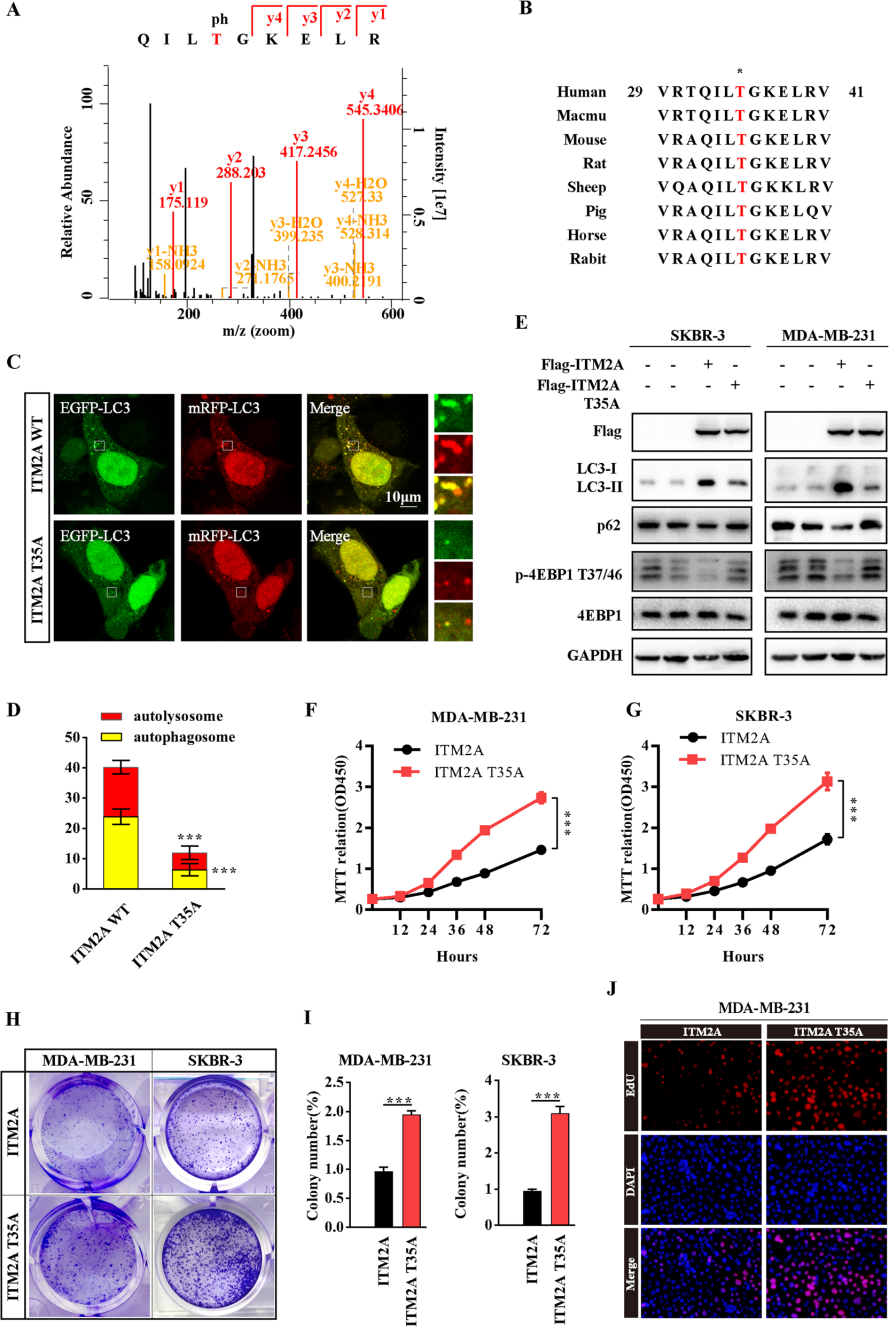
ITM2A is phosphorylated at T35 and the phosphorylation status of ITM2A contributes to breast cancer proliferation.(**a**) The mass spectrum showed that ITM2A was phosphorylated at T35. (**b**) Amino acid sequences around the threonine 35 residue in ITM2A across different species. The asterisk at the top, represents the threonine residue that is conserved across species. (**c** and **d**) Representative confocal images of mRFP-GFP-LC3 puncta in SKBR-3 cells transfected with ITM2A wild-type and T35A mutant vector. The numbers of cells showing accumulation of yellow or red puncta were quantified (*n* = 10). (**e**) Western blotting analysis of SKBR-3 and MDA-MB-231 cells transfected with empty vector, ITM2A expression vector and ITM2A T35A mutant vector using the indicated antibodies. (**f** and **g**) The growth curve of MDA-MB-231 and SKBR-3 cells transfected with wild-type ITM2A and T35A mutant plasmids. (**h** and **i**) Colony formation assays were performed for MDA-MB-231 and SKBR-3 cells transfected with wild-type ITM2A and T35A mutant plasmids. (**j**) DNA synthesis of MDA-MB-231 cells transfected with wild-type ITM2A and T35A mutant plasmids was assessed by EdU assays. Data are presented as the mean ± SD. Two-tailed Student’s t-test was used. *P <  0.05; **P <  0.01; ***P <  0.001

To explore the post-translational modification of ITM2A, we collected the ITM2A immunoprecipitate and sent it for mass spectrometry analysis. The results indicated that ITM2A was highly phosphorylated at T35 (Fig. 3a). After sequence alignments, we found that the amino acid sequence surrounding T35 is highly conserved in ITM2A among multiple species (Fig. 3b). We then replaced T35 with alanine (A) to generate a nonphosphorylatable mutation of the ITM2A protein and texted whether T35 contributed to autophagy induction in human breast cancer cells. The results revealed that the T35A mutation of ITM2A showed a significantly lower number of red-only puncta compared with wild-type ITM2A (Fig. 3c and d). Moreover, the high level of LC3-II was markedly decreased, whereas the level of p62 was increased after T35A mutation transfection compared with wild-type ITM2A in both SKBR-3 and MDA-MB-231 cells (Fig. 3e). We then examined the effect of ITM2A T35A on mTOR activity and the results showed that the T35A mutation abolished the inhibitory effect of ITM2A on mTOR activity, as indicated by the reversed level of p-4EBP1 at T37/46 (Fig. 3e). These data indicated that ITM2A is phosphorylated at T35 and that the phosphorylation status of ITM2A is required for autophagy induction. We then examined the effects of ITM2A T35A mutation on breast cancer proliferation. The results showed that the ITM2A T35A mutation exhibited higher growth, colony formation and DNA synthesis ability compared with those of wild-type ITM2A (Fig. 3f-j).

### ITM2A is phosphorylated by HUNK

**Fig. 4:**
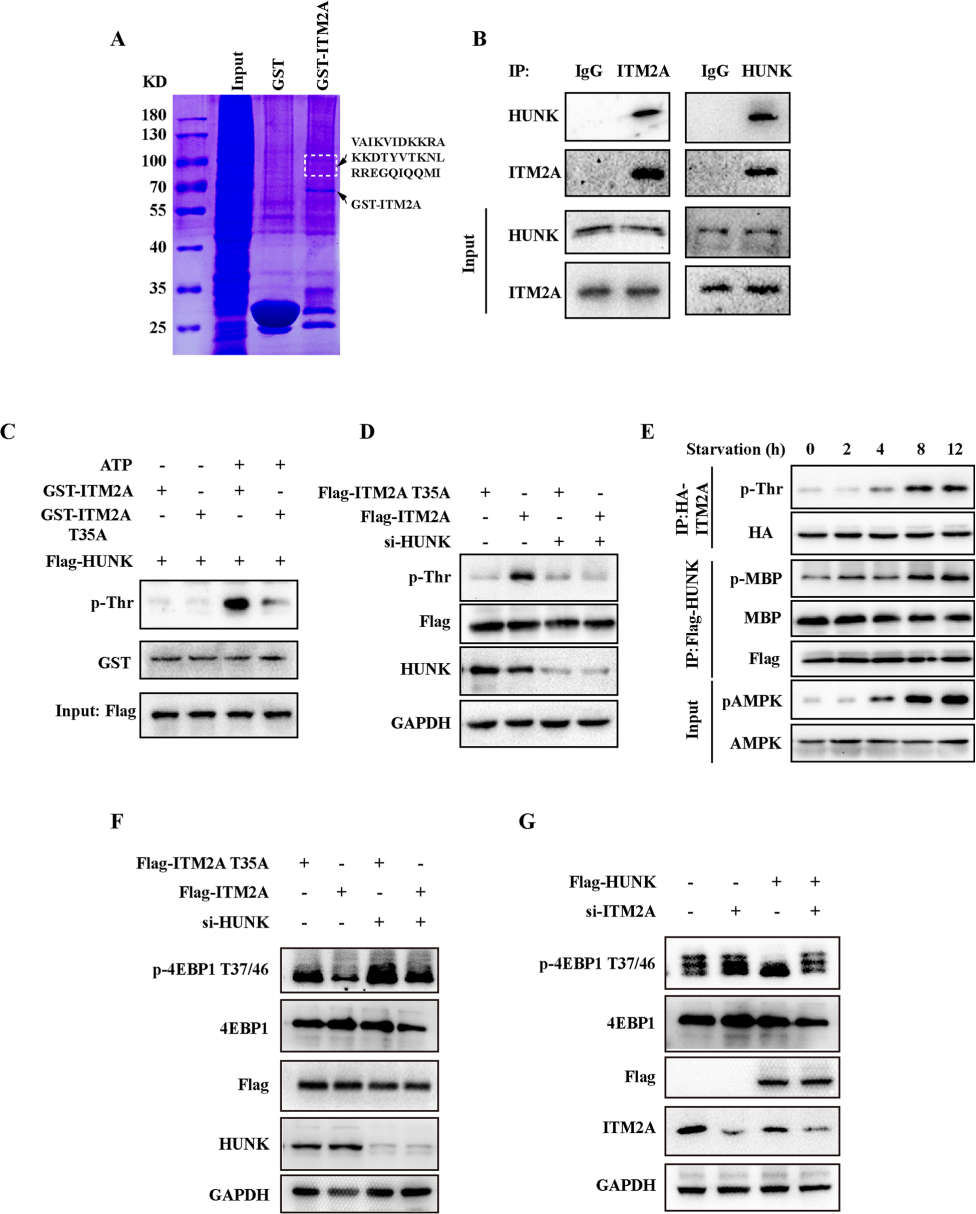
ITM2A interacts and is phosphorylated by HUNK. (**a**) Coomassie brilliant blue staining showed that the peptide “VAIKVIDKKRAKKDTYVTKNLRREGQIQQMI” existed in the 70 kDa–100 kDa band. (**b**) Co-immunoprecipitation showed the interaction between ITM2A and HUNK in HEK293T cells. (**c**) Flag-HUNK were obtained from the immunoprecipitate in HEK293T cell lysates using Flag antibody. The Flag-HUNK was incubated with the in vitro purified GST-ITM2A WT or T35A protein at 30 °C for 30 min with or without ATP addition, and then the reaction products were subjected to western blotting. (**d**) Flag-ITM2A was obtained from the immunoprecipitate in SKBR-3 cell lysates with or without HUNK knockdown using Flag antibody. Total phosphorylated threonine was detected in the western blotting assay. (**e**) SKBR-3 cells transfected with HA-ITM2A or Flag-HUNK were starved for the indicated times, and HA-ITM2A was immunoprecipitated using HA antibody and used for western blotting analysis. Purified Flag-HUNK was used in the in vitro kinase assay using purified MBP protein as a substrate. (**f**) SKBR-3 cells were transfected with wild-type ITM2A and its T35A mutant simultaneously with or without HUNK siRNA co-transfection for 48 h. Cell lysates were obtained and used for detection by western blotting with the indicated antibodies. (**g**) HEK293T cells were transfected with empty vector and Flag tagged HUNK simultaneously with or without ITM2A siRNA co-transfection for 48 h. Cell lysates were obtained and used for detection by western blotting assay with the indicated antibodies

We then set out to investigate the potential kinase(s) that could phosphorylate ITM2A. For this analysis, we carried out a GST pull-down assay using the fused protein GST-ITM2A, and the results revealed a peptide sequence “VAIKVIDKKRAKKDTYVTKNLRREGQIQQMI”, which represented the possibility of Up-regulated Neu-associated Kinase (HUNK), a serine/threonine kinase in the GST-ITM2A precipitate (Fig. 4a). HUNK was reported to promote autophagy and breast cancer survival in response to lapatinib [25]. A high level of HUNK was found in HER2-E subtype breast cancers and was significantly correlated with overall survival and promoted HER2 induced mammary tumorigenesis in vivo [26]. According to our findings, which showed a significant prognostic effect of ITM2A in HER2-E subtype breast cancer patients, it presents huge possibility that HUNK could phosphorylate ITM2A. We first texted whether HUNK interacted with ITM2A, and as expected, the results showed that HUNK existed in the immunoprecipitate of ITM2A as tested by a specific antibody in HEK293T cells (Fig. 4b). In the in vitro kinase assays, GST-ITM2A WT and ITM2A T35A were incubated with Flag-HUNK immunoprecipitate with or without ATP. The results revealed that GST-ITM2A WT readily detected phosphorylated threonine in the presence of ATP. However, phosphorylated threonine was barely detected in the GST-ITM2A T35A group (Fig. 4c). Furthermore, we detected phosphorylated threonine in ITM2A transfected SKBR-3 cells but barely in ITM2A T35A transfected cells. We found that the phosphorylated threonine in wild-type ITM2A transfected SKBR-3 cells was significantly decreased after HUNK knockdown (Fig. 4d). Indicating that HUNK is the main kinase that phosphorylates ITM2A threonine residues. We also found that the threonine phosphorylation level of ITM2A was much more enhanced in EBSS starvation for 2 h, 4 h, 8 h and 12 h and was accompanied by the increased activity of HUNK, as indicated by the phosphorylation of myelin basic protein (MBP) (Fig. 4e).

We next investigated the roles of HUNK and ITM2A on the regulation of mTOR activity. To do this, we transfected wild-type ITM2A and its T35A mutant simultaneously with or without HUNK siRNA co-transfection in SKBR-3 cells. The results indicated that HUNK knockdown decreased mTOR activity either co-transfected with wild-type ITM2A or T35A mutant, however, it was much more robust in wild-type ITM2A transfected cells (Fig. 4f). We also confirmed that ITM2A knockdown increased the level of p-4EBP1 at T37/46 in HEK293T cells. Moreover, the mTOR activity in HUNK overexpressing cells was much higher compared with that of ITM2A siRNA co-transfected cells (Fig. 4g). Collectively, our data indicated that ITM2A is phosphorylated by HUNK at T35.

## Discussion

Recently, ITM2A has been identified as a novel tumor suppressor in epithelial ovarian cancer [20]. However, the biological role of ITM2A in breast cancer has never been elucidated. Through genome-wide expression analysis, we found that the mRNA level of ITM2A was 7.40-fold down-regulated in breast cancer tissues compared with the level in normal tissues. We also confirmed that ITM2A expression retarded growth and reduced colony formation in breast cancer. Moreover, we found that ITM2A expression significantly correlates with age, TNM classification and tumor stage. ER and PR states are broadly applied in the clinic to predict the response to hormone therapy, and HER2 status is utilized to predict the response to therapeutic regimens [27]. We demonstrated that ITM2A expression significantly correlates with PR status, but not with ER status and HER2 level, suggesting that it could be more effective to use hormone therapy for breast cancer patients with PR positive status and low ITM2A expression. These findings provide more information for clinicians to diagnose and select the appropriate treatment using parameter of ITM2A.

Dysfunction of the autophagy pathway has been linked to various human cancers, either enhancing or preventing tumorigenesis depending on the tumor type or context [28]. Autophagy is activated in rapidly growing tumors in regions of hypoxia and/or nutrient deprivation, such as pancreatic cancer, which requires autophagy for tumor growth [29]. In this context, genetic or pharmacologic inhibition of autophagy could lead to increased reactive oxygen species, elevated DNA damage, and a metabolic defect leading to the regulation of cell proliferation retardation or cell death [30]. Autophagy can also promote the survival of many cancers in the case of the stress of therapies (chemotherapy, radiotherapy, and targeted agents) and thus promote therapeutic resistance [31]. However, autophagy was initially considered to be a process that suppressed malignant transformation with a role in regulating the homeostasis of cellular proteins, lipids, and organelles [30]. Genetically engineered liver-specific autophagy related 7 (L-ATG7) or L-ATG5 knockout (KO) mice exhibit increased development of spontaneous liver tumors [32, 33]. Aberrant PI3K/AKT pathway activation via activating PI3K mutations, amplification of genes encoding HER2, EGFR and AKT, BCR-ABL translocation or PTEN loss leads to decreased autophagy in many tumor cells largely through mTOR activation [34]. Discovery of single copy loss of the BECN1/Beclin1 gene, also known as the mammalian ortholog of yeast ATG6, in ovarian cancers provided more direct evidence of the tumor-suppressing properties of autophagy. In this study, we also highlight the tumor suppression role of autophagy, particularly ITM2A overexpression induced autophagy, in breast cancer proliferation.

AMPK and mTOR are the two central modulators of autophagy regulation [8]. The roles for AMPK in autophagy induction and the inhibitory function of mTOR complex 1 (mTORC1) in autophagy are well established [35, 36]. In the context of autophagy induction, our data showed that ITM2A has a limited effect on AMPK activity. However, overexpression of ITM2A significantly inhibits activation of mTOR, as indicated by the reduced threonine phosphorylated 4EBP1 at both the 37/46 sites. As a well characterized mTORC1 target, 4EBP1 binds and inactivates eukaryotic translation initiation factor 4E (eIF4E) and then inhibits the initiation of protein translation. Phosphorylation of 4EBP1 by mTORC1 promotes the dissociation of eIF4E from 4EBP1 and blocks its inhibitory effects on the initiation of protein translation [37]. As part of the PI3K/AKT signaling network, mTOR is a powerful stimulator of cell proliferation [38]. As ITM2A is downregulated in breast cancer, the inhibitory effect of ITM2A to mTOR could be abolished. It was previously reported that a large amount of GFP-ITM2A was detected in the perinuclear region and mainly localized in the Golgi, with a tiny part of the GFP-ITM2A colocalized with lysosomes [39]. mTORC1 is known to recruit to lysosomes and is activated by the small GTPase RHEB, and RHEB activates lysosome-localized mTORC1 at the Golgi-lysosome contact site [40]. It is possible that ITM2A blocks mTORC1 activation by interacting with RHEB and inhibiting the binding of RHEB to mTORC1, which however, requires further verification.

HUNK was discovered in a screen to identify protein kinases in mammary epithelial tumor cells derived from several genetically engineered mouse models (GEMMs) [41]. HUNK is an ~ 80 kDa protein with three domains: a catalytic kinase domain, an SNF1 homology domain (SNH domain) and a ubiquitin-associated domain (UBA domain) [42]. HUNK has been shown to be highly expressed in HER2 positive breast cancer cell lines and to supporte the survival of HER2/neu-positive tumor cells [25]. However, the physiological role of HUNK has been largely elusive, and there are no known HUNK substrates that have been identified. In this study, we first linked the role of HUNK and ITM2A in regulating autophagy and demonstrated that ITM2A is a substrate of HUNK. We further identified that HUNK phosphorylated ITM2A at T35. Although we demonstrated the tumor suppression role of ITM2A in human breast cancer patients, more future studies are needed to underline the roles of the HUNK/ITM2A axis.

## Conclusions

In summary, we identified significantly down-regulated expression of ITM2A that was associated with poor prognosis in human breast cancer patients, especially in HER2-E subtype breast cancer patients, through bioinformatics analysis. We also confirmed the biological role of ITM2A in human breast cancer cells of promoting autophagy and that HUNK phosphorylated ITM2A at T35. Our data suggest that ITM2A could be a valuable prognostic marker and promising diagnostic and therapeutic target for breast cancer in the future.

## Additional file

Additional file 1: **Figure S1.** The prognostic impact of ITM2A on disease outcome in breast cancer patients with different ER and nodal statuses. **Figure S2.** The prognostic impact of ITM2A on disease outcome in different breast cancer patient subtypes. **Figure S3.** ITM2A knockdown impairs autophagy. (DOCX 1063 kb)

## Data Availability

All data generated or analyzed during this study are included in this published article and supplementary information files.

## Abbreviations

AMPK: Adenosine 5′-monophosphate (AMP)-activated protein kinase
BC: Breast cancer
ER: Estrogen receptor
HER2: Human Epidermal Growth Factor Receptor 2
HUNK: Up-regulated Neu-associated Kinase
IHC: Immunohistochemical
ITM2A: Integral membrane protein 2A
MR: Metastatic relapse
mTOR: The mammalian target of rapamycin
MTT: 3-[4,5-dime-thylthiazol-2-yl]-2,5 diphenyl tetrazolium bromide
PR: Progesterone receptor
RHEB: Ras homolog enriched in brain
Ser: Serine
TCGA: The Cancer Genome Atlas
Thr: Threonine
