# Supplementary material for: Integral membrane protein 2A inhibits cell growth in human breast cancer via enhancing autophagy induction
Source: Cell Commun Signal. 2019 Aug 22;17:105. doi: 10.1186/s12964-019-0422-7 (PMC6704577; doi:10.1186/s12964-019-0422-7)
Supplement: Supplementary file 1 — Figure S1. The prognostic impact of ITM2A on disease outcome in breast cancer patients with different ER and nodal statuses. Figure S2. The prognostic impact of ITM2A on disease outcome in different breast cancer patient subtypes. Figure S3. ITM2A knockdown impairs autophagy. (DOCX 1063 kb) [file 12964_2019_422_MOESM1_ESM.docx]

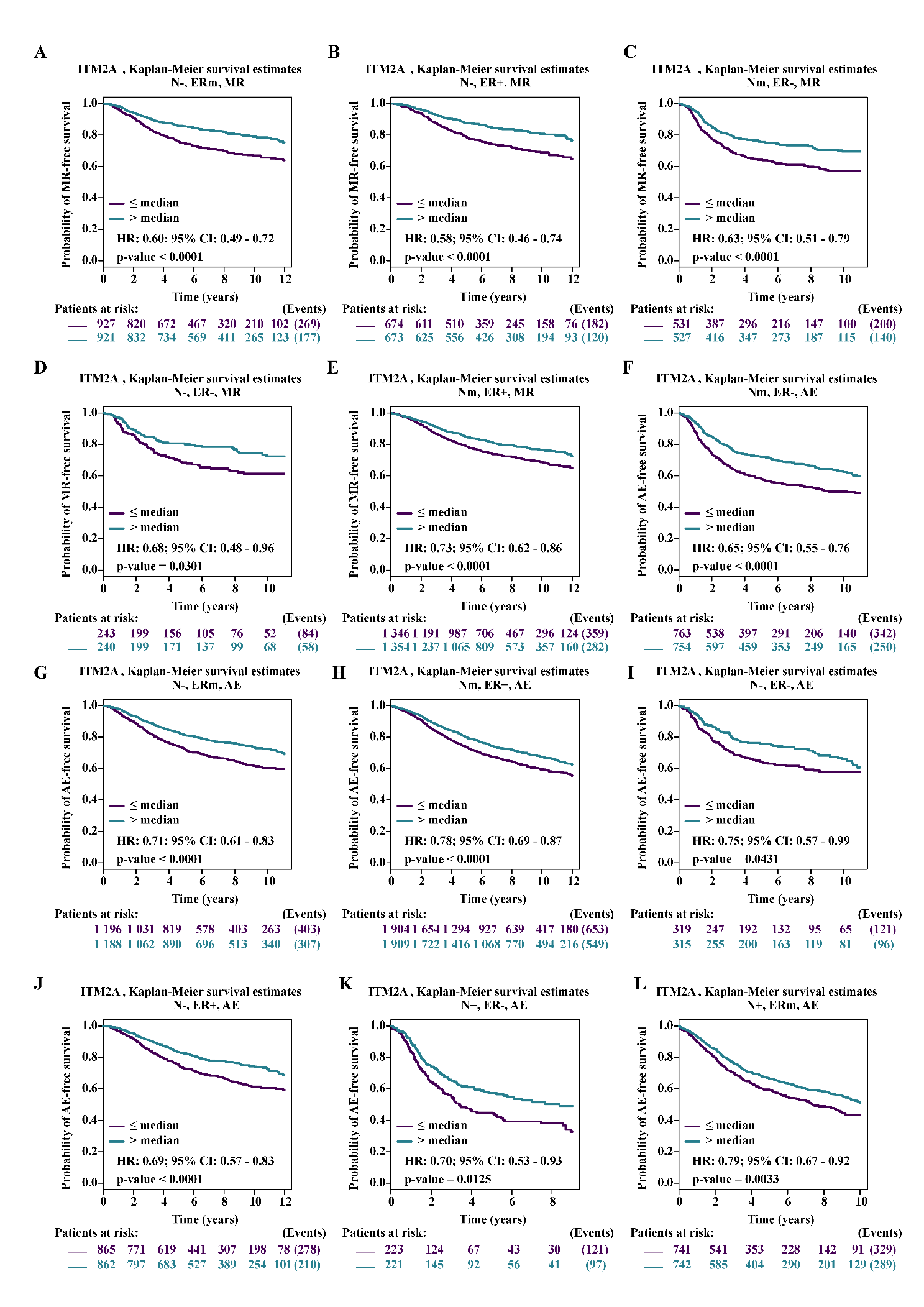


**Figure S1.** The prognostic impact of ITM2A on disease outcome in breast cancer patients with different ER and nodal statuses.

(**A-E**) Kaplan-Meier curves in metastatic relapse free (MR) situation for ITM2A in N-, ERm (A), N-, ER+ (B), Nm, ER- (C), N-, ER- (D) and Nm, ER+ (E) patients. (**F-L**) Kaplan-Meier curves in any event (AE) situation for ITM2A in Nm, ER- (F), N-, ERm (G), Nm, ER+ (H), N-, ER- (I), N-, ER+ (J), N+, ER- (K) and N+, ERm (L). Any event refers to first pejorative event represented by any relapse or death.


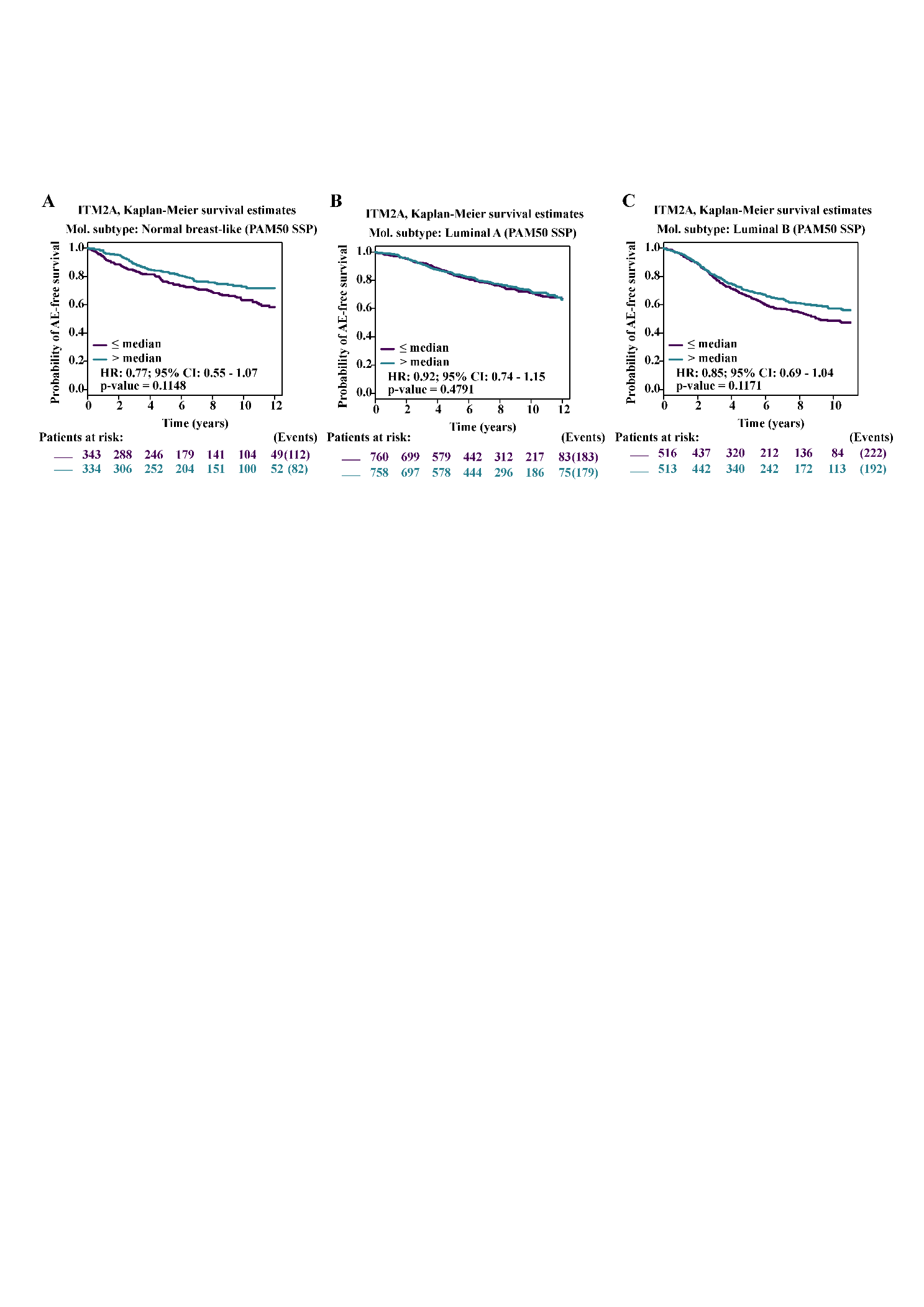


**Figure S2.** The prognostic impact of ITM2A on disease outcome in different breast cancer patient subtypes.

Kaplan-Meier survival curves for ITM2A within the breast cancer molecular subtypes, including normal breast-like (A), luminal A (B) and luminal B (C) subtypes.


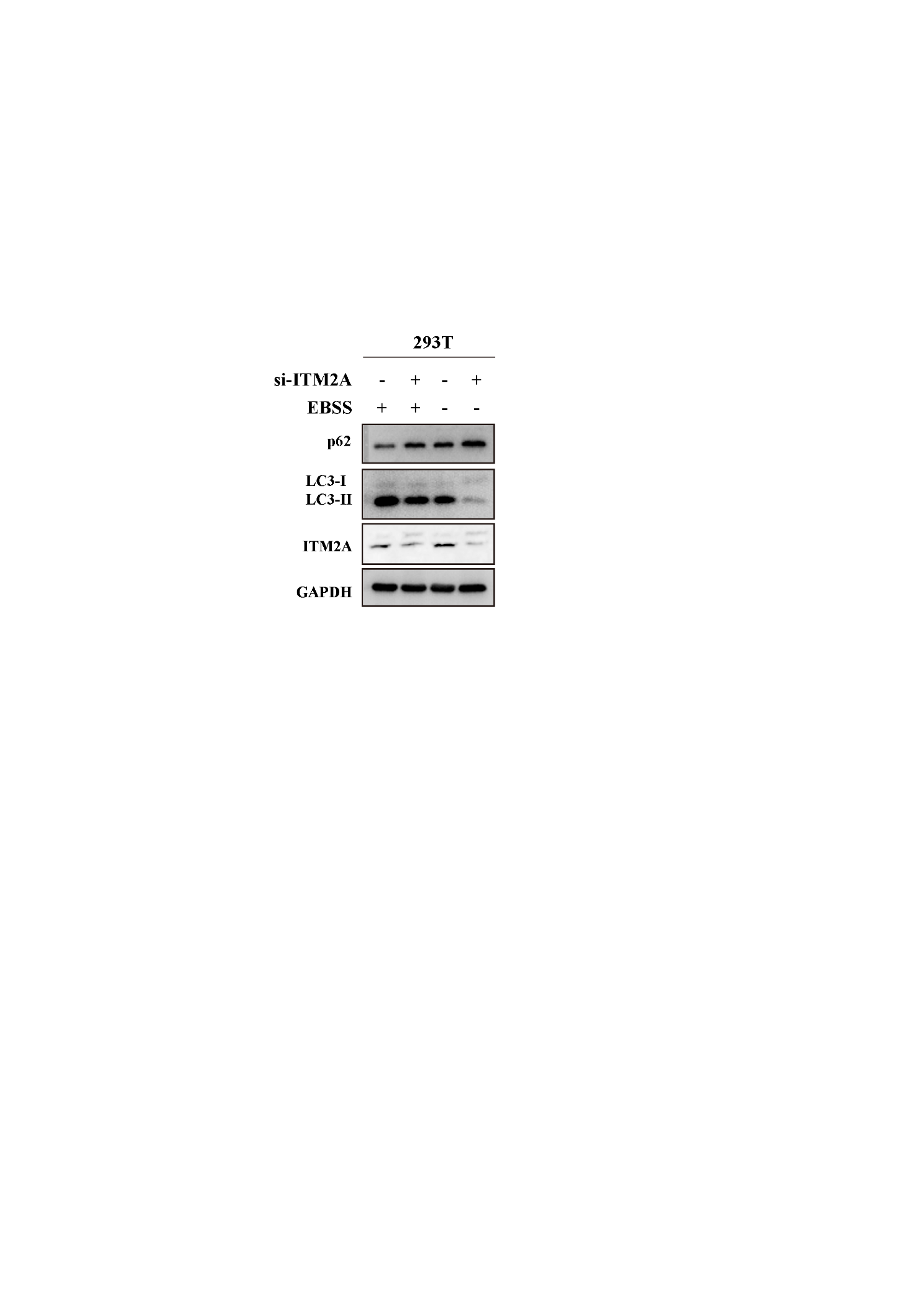


**Figure S3.** ITM2A knockdown impairs autophagy.

Western blotting analysis in HEK293T cells transfected with scramble RNA and ITM2A small interfering RNA using indicated antibodies.
